# The Effect of Thyrotropin-Releasing Hormone and Antithyroid Drugs on Fetal Thyroid Function

**DOI:** 10.3390/children8060454

**Published:** 2021-05-28

**Authors:** Nikolaos Vrachnis, Orestis Tsonis, Dionisios Vrachnis, Nikolaos Antonakopoulos, George Paltoglou, Stavroula Barbounaki, George Mastorakos, Minas Paschopoulos, Zoi Iliodromiti

**Affiliations:** 1 Third Department of Obstetrics and Gynecology, National and Kapodistrian University of Athens Medical School, Attikon Hospital, 157 72 Athens, Greece; antonakopoulos2002@yahoo.gr; 2 Vascular Biology, Molecular and Clinical Sciences Research Institute, St George’s University of London, London SW17 0RE, UK; 3 Department of Obstetrics and Gynecology, University of Ioannina Medical School, University Hospital of Ioannina, 455 00 Ioannina, Greece; orestis.tsonis@gmail.com (O.T.); mpaschop@gmail.com (M.P.); 4 Department of Clinical Therapeutics, School of Medicine, National and Kapodistrian University of Athens, Alexandra Hospital, 157 72 Athens, Greece; dionisis_94@hotmail.com; 5 Endocrinology Unit, 2nd Department of Obstetrics and Gynecology, National and Kapodistrian University of Athens Medical School, Aretaieio Hospital, 157 72 Athens, Greece; gpaltoglou@gmail.com (G.P.); mastorakg@gmail.com (G.M.); 6 National Merchant Marine Academy of Aspropyrgos, 193 00 Aspropyrgos, Greece; sbarbounaki@yahoo.gr; 7 Department of Neonatology, National and Kapodistrian University of Athens Medical School, Aretaieio Hospital, 157 72 Athens, Greece; ziliodromiti@yahoo.gr

**Keywords:** thyroid, fetus, TRH, antithyroid drugs, feto-maternal interaction

## Abstract

A euthyroid pregnant woman will normally have a fetus that displays normal fetal development. However, studies have long demonstrated the role of T3 (Triiodothyronine), T4 (Thyroxine), and TSH (Thyroid Stimulating Hormone) and their degree of penetrability into the fetal circulation. Maternal thyrotropin-releasing hormone (TRH) crosses the placental site and, from mid-gestation onward, is able to promote fetal TSH secretion. Its origin is not only hypothalamic, as was believed until recently. The maternal pancreas, and other extraneural and extrahypothalamic organs, can produce TRH variants, which are transported through the placenta affecting, to a degree, fetal thyroid function. Antithyroid drugs (ATDs) also cross the placenta and, because of their therapeutic actions, can affect fetal thyroid development, leading in some cases to adverse outcomes. Furthermore, there are a number of TRH analogues that share the same properties as the endogenous hormone. Thus, in this narrative review, we highlight the interaction of all the above with fetal growth in uncomplicated pregnancies.

## 1. Introduction

Over the years, a large number of studies have been conducted focusing on maternal and fetal thyroid function and their interaction [1,2,3,4]. Throughout a normal pregnancy, the fetal thyroid gland develops and, as early as the 10th week of gestation, thyroid follicles and thyroxine (T4) fetal synthesis are observed [5]. By the 11th week of gestation, fetal T4 is detectable, which then gradually increases, although little is as yet known about the molecular mechanisms underlying the initiation of human thyroid function. The role of thyroid hormones and their impact on fetal thyroid function, however, have been well documented over time [6].

The fetal environment was previously considered impermeable to thyroid hormones due to the presence of degradative enzymes in the placental site [7]. However, a fetal thyroid is unable to secrete its own thyroid hormones until the 18th week of gestation and is dependent on the maternal component to achieve normal thyroid function and development [8,9,10]. Iodine and T4 are transferred to the fetus during a normal pregnancy [1,11]. It is of importance to underline the significance of T4 transfer from mother to fetus, since this action throughout pregnancy strongly affects both fetal thyroid function and fetal development.

Although there has been extensive scientific research into thyroid hormone transport providing essential information on the interaction between maternal and fetal components, the investigations have so far failed to determine what may be the effect of thyrotropin-releasing hormone (TRH) and antithyroid drugs (ATDs) on the fetal thyroid gland, given that the placenta shows a certain degree of permeability [10,12].

TRH was the name of the first tripeptide pyroglutamyl–histidyl–proline amide (pyro-Glu–His–Pro amide) given by Guillemin and Schally in the 1960s, it being the first hypothalamic-releasing factor that was isolated and structurally characterized [13]. During the 1980s, studies in mammals revealed that TRH is produced by hypothalamic paraventricular neurons as well as by other extra-hypothalamic brain areas, whereas TRH-like peptides originate in extraneural tissues, such as the pancreas and gastrointestinal tract [13]. These TRH-like peptides differ from authentic TRH due to the fact that the amino acid histidine of authentic TRH is replaced by neutral or acidic amino acids, such as glutamic acid, glutamine, asparagine, phenylalanine, leucin, valin, tyrosine, and aspartic acid, this accounting for the different degrees of penetrability of these biological potent TRH variants [14].

TRH, after being activated in the anterior pituitary, binds to TRH receptors, stimulating the synthesis and the secretion of the TSH β-subunit. Following its binding to the TSHR, TSH mediates a number of effects on thyroid hormone metabolism, some being iodotyrosine synthesis, Tg synthesis, iodine trapping, and hormone release.

In this narrative review, we analyze recent data on and new insights into the effect of thyrotropin-releasing hormone and ATDs on fetal thyroid function in uncomplicated pregnancies.

## 2. Materials and Methods

For our literature review, we searched the MEDLINE, PubMed, and EMBASE databases. Articles focusing on the effect of TRH and ATDs on fetal thyroid function were included. We used the following keywords either alone or in combination: fetal thyroid function; thyrotropin releasing hormone; thyroid hormones; maternal fetal thyroid hormone interaction; thyrotropin releasing hormone analogues; and antithyroid drugs. Only papers in the English language were included in this review. Scientific evidence addressing abnormal pregnancies, as, for example, FGR (fetal growth restriction) or preeclamptic cases, were excluded. Moreover, this review does not focus on placental hormones that potentially affect fetal thyroid function except in reference to those reviewed, such as placental TRH. Lab model information was also included in order to clarify as far as possible the physiology of fetal thyroid function in a normal pregnancy.

## 3. The Effect of Thyrotropin-Releasing Hormone (TRH) in the Development of Fetal Thyroid Function

Maternal TRH demonstrates high penetrability into the fetal environment and is present in the fetal brain since the early stages of gestation, long before the fetal hypothalamus is developed [15,16]. From conception until the fetal hypothalamic–pituitary axis (HPA) is autonomous, the maternal thyroid hormones, including T3, T4, and iodine, facilitate normal fetal thyroid development [3,6]. A large number of studies in both humans and other species support the penetrability of maternal T4 through the placenta during virtually the entire period of pregnancy, but not vice versa [15,17,18]. Nevertheless, this transport facilitates only small amounts of T4 from the maternal to the fetal compartment. The latter could possibly explain sporadic hypothyroidism in human neonates despite normal maternal thyroid function, since maternal thyroid hormones are not sufficient to prevent it [15,17,18]. In the event of a substantial elevation in maternal T4 concentrations, as, for example, if the mother has thyroid hormone resistance, there may be excessive T4 transfer, which can result in an adverse outcome, such as fetal loss [19].

**Figure 1:**
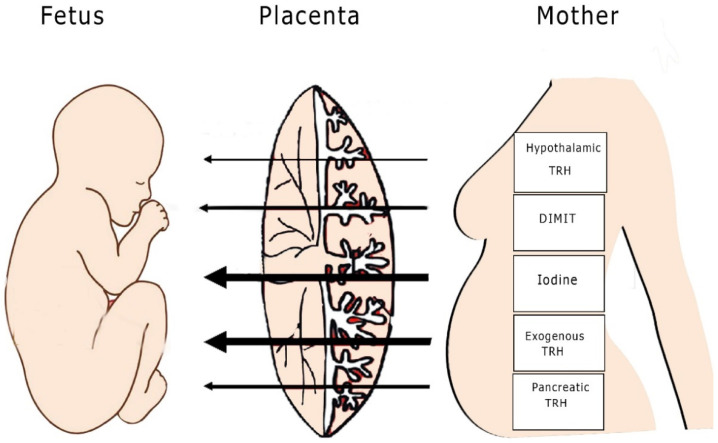
Penetrability of thyroid hormones. The thickness of the arrows reflects the level of penetrability of each element (TRH: thyrotropin-releasing hormone; DIMIT: 3′,5′-dimethyl,5-isopropyl thyronine).

Beta human chorionic gonadotropin (βHCG) is produced by the placenta and shows similarities in its structure to TSH. Although of similar structural homology, the bioactivity of βHCG compared to TSH is fairly low. TSH-like activity exerted by placental chorionic gonadotropin has a negligible effect on the fetal hypothalamic–pituitary–thyroidal axis and fetal thyroid function [20,21,22] (Figure 1).

The synthesis of thyroid hormone in the human fetus is mainly regulated by the availability of iodine in the fetal environment [23]. Radioactive signaling techniques have shown that in many species, including rats, sheep, and humans, iodine is transported by the placenta to the fetus, thereby enabling normal fetal thyroid function [15]. However, iodine deficiency during pregnancy results in perinatal hypothyroidism [15]. Relevant data have shown that when radioiodine was used for maternal hyperthyroidism, the treatment caused fetal hypothyroidism. Based on this observation, it is evident that the placenta is highly permeable to iodine. Increased iodine intake during pregnancy should be avoided due to its deleterious effects on the fetal thyroid since it may impede thyroid hormone production (the Wolff–Chaikoff effect) [24].

The fetal hypothalamus and pituitary gland mature approximately at 20 weeks, during mid-term pregnancy, but maternal TRH can be measured in the fetal circulation earlier (approximately at 12 weeks), thus indicating that TRH can cross the placenta [16]. Although some studies suggest that this hormone does not affect the maturation of the fetal hypothalamic–pituitary axis (HPA), there is some uncertainty as to whether maternal TRH affects fetal TSH secretion. Hence, the possibility of an indirect effect of maternal TRH on fetal thyroid function needs to be further explored [15]. Studies on anencephalic human fetuses and studies in animals that underwent surgical removal of the maternal and fetal hypothalamus or to which TRH antibodies were administered demonstrated that the role of TRH is probably auxiliary rather than major, since no fetal hypothyroidism was noted [15] (Figure 1).

In animal studies where TRH deprivation was effected through the use of antibodies, no abnormalities in TSH secretion were noted. Though the role of TRH is still unclear, it seems that fetal thyroid function is not affected directly by the absence of TRH in the fetal circulation. Independently of its origin, the role of TRH is yet to be clarified [25]. Moreover, studies involving cordocentesis demonstrated that TRH can affect fetal TSH secretion after 37 weeks of gestation. This shows that normal fetal hypothyroidism is more susceptible at term given that its physiology at that point follows a different pattern [26]. Furthermore, TRH administered in a single dose could potentially facilitate pulmonary maturation in an augmented way, as achieved by corticosteroids [27]. The non-halogenated thyronine 3′,5′-dimethyl,5-isopropyl thyronine (DIMIT) acts as a thyroid analogue of T3 and suppresses both TSH and TRH, lowering their concentrations in both the maternal and the fetal circulation [15]. DIMIT’s action does not seem to considerably affect fetal thyroid function, although the placenta is fairly permeable to this drug [9].

TRH levels in the maternal circulation are higher than those of non-pregnant women due to downregulation of TRH-degrading activity in the serum as pregnancy progresses [28]. However, this fact does not seem to add more information to what is already known about the role of TRH in fetal thyroid function. Since its contribution to fetal development is as yet unclear, further studies are needed to conclusively elucidate its function [28,29].

## 4. The Effect of ATDs in the Development of Fetal Thyroid Function

The thionamide ATDs carbimazole (CBZ) and methimazole (MMI), to name the most common ATDs in use, cross the placenta [30]. Studies on animal models confirm that these drugs pass through the placental barrier and have an effect on the development of fetal thyroid function [15]. These drugs, which are extensively used to treat cases of Graves’ disease and toxic nodular goiter, are linked to a higher incidence of fetal goiter (10%) and neonatal hypothyroidism when they are used during pregnancy [15,31].

The use of small doses of prophylthiouracil (PTU), another thionamide drug, appears to have a lesser effect on fetal thyroid function [32,33]. Prophylthiouracil is effective in treating maternal hyperthyroidism during pregnancy, but induced fetal thyroid hypothyroidism is inevitable if doses are not kept to a minimum [15]. Doses of PTU below 300 mg seem to be safe for fetal function. However, regarding doses above this limit, there are reports that link the drug to adverse fetal thyroid dysfunction, although the evidence is not consistent [15]. For example, in a case of a twin pregnancy, only one of the two fetuses was affected by the drug, leading to fetal goiter [15] (Table 1).

Since there is no scientific evidence that favors the use of any particular thionamide ATD, safety in their usage depends on the dosage. This group of drugs shows a fast transfer into the fetal circulation [15]. The placenta is more permeable to MMI than to prophylthiouracil; hence, there is an increased risk for fetal hypothyroidism with use of this ATD [15]. One study has demonstrated that PTU displays a lower degree of transplacental passage compared to MMI [34]. Data meta-analysis showed that both MMI and PTU carry the same risk for development of neonatal goiter and hypothyroidism [34]. This meta-analysis revealed no actual differences in pregnant women treated with PTU or MMI where the prevalence of neonatal hypothyroidism was concerned; it is thus reasonable to assume that choice of any specific ATD is less important than maintenance of stability of maternal hormonal status during medication [35,36]. Nevertheless, according to the guidelines of both the American Thyroid Association and the European Thyroid Association, PTU can be used only in the first trimester due to increased fetal hepatotoxicity. On the other hand, it has been well-documented that MMI is associated with teratogenicity in the first trimester and, therefore, PTU is preferred [37,38].

Although the above-mentioned drugs are designed to restore normal thyroid function in the mother, cumulative data have so far demonstrated that fetal thyroid function is affected primarily by ATDs rather than by maternal thyroid hormones [39]. Current studies demonstrate that the minimum doses of ATDs should be used in order to achieve borderline normal maternal thyroid function, as indicated by an upper limit free-T4 to total-T4 ratio, so that they will not pose a threat to the majority (90%) of neonates [39]. On the other hand, adverse neonatal outcomes may well arise from untreated cases of maternal hypothyroidism. Hence, if risk-free treatment options are not available, clinicians should perform evidence-based management in order to decide upon the most suitable and individualized approach for each patient [40,41].

## 5. TRH Analogues, Synthetic Drugs, and Their Action in the Development of Fetal Thyroid Function

Exogenous TRH can also cross the placental barrier, as this has been demonstrated in a human study revealing that exogenous TRH administration increases cord blood TSH serum levels within 20 min [42]. Another study in pigs reported that immune-reactive exogenous TRH was detected in the fetal circulation within 5 min of administration [15]. The majority of studies conclude that TRH administration, from mid-pregnancy onward, leads to an increase in TSH levels in the fetal circulation only minutes after the ejection [15]. Other studies have reported that placental TRH has the same immunological and chromatographic properties as those of synthetic TRH, thus possibly explaining the crucial role of placental TRH in balancing fetal TSH concentrations [43,44]. Nevertheless, it is still unknown whether placental TRH actions are similar to those of synthetic TRH, since although chemically identical, their true physiological role is yet to be determined [44]. This phenomenon highlights the autonomy of the fetal thyroid gland and its means of counteracting an increase of TRH [45].

However, different variants of TRH originating in the maternal hypothalamus or maternal pancreas, or given via exogenous administration, have different rates of penetrability into the placenta [46]. Studies on rats demonstrated high penetrability of maternal pancreatic TRH but not of maternal hypothalamic TRH [46]. Although during the first weeks of life only maternal pancreatic TRH was found to cross the placenta, from then onward, maternal TRH originating from the maternal (extrahypothalamic) forebrain and brain stem, gastrointestinal tract and liver, spleen, kidney, and heart was also detected [44]. It seems that maternal exogenous TRH administration crosses the placental site and achieves thyroid stimulation, resulting in fetal TSH secretion, as has been indicated by examination of human blood cord samples [27,42]. It is of note that administration of TRH to the mother is effective in elevating fetal T3 levels, while the treatment has not been shown to repress the surge of TSH and T3 shortly after birth [47].

The TRH stimulation test, which shows the exogenous administration of TRH in the maternal circulation, has long demonstrated sufficient TRH penetrability through the placenta in a variety of species, e.g., rats, lambs, and rhesus monkeys. Any maternal TRH form does cross the placenta, since after performing the test a surge of TSH synthesis in the human fetal circulation was observed in many studies [42,48]. It was shown that TRH administration to a pregnant rhesus monkey induced a greater response in fetal plasma TSH than in maternal TSH, while, subsequently, there were higher increments in concentrations in the fetus of plasma T4 and T3 concentrations than there were in the mother. Also noted was a significant increase in plasma prolactin in both the mother and fetus following administration of TRH to either the mother or fetus, with the increase in plasma PRL being far higher in the mother than in the fetus. It was also demonstrated that TRH is able to cross the primate placenta in both directions, namely, maternal to fetal or fetal to maternal [49]. In a study on rats, circulating TRH was proven to be of maternal pancreatic origin rather than hypothalamic [46]. This emphasizes the complicated physiologic patterns that TRH circulation follows in which even different homologues of the hormone result in different concentrations of the hormone in the fetal circulation [46].

In addition, research shows that TRH, which actually crosses the placental barrier, is not of maternal or fetal hypothalamic origin. The hypothesis of maternal extraneural TRH crossing the placenta has been confirmed through various studies and mentioned above. Nevertheless, no clear evidence of the penetrability of maternal hypothalamic TRH has been documented or measured. Complicated mechanisms functioning in the placental site are yet to be fully clarified; elucidation of these is necessary in order to demonstrate whether, and if so to what extent, maternal TRH can cross the placental barrier [43,46]. In Table 1, various types of TRH and ATDs listed according to their penetrability are presented. Interestingly, maternal hypothalamic and exogenous TRH, although of the same molecular weight, display different degrees of penetrability. It is possible that these elements, although chemically identical, possess different biological capacities. Immunological determinants of these elements demonstrate altered biological potency, as has been revealed in some studies [43]. This may account for the fact that the penetrability of ATDs is not determined solely by their molecular weights.

## 6. Conclusions

The role of TRH and ATDs in the development of fetal thyroid function is complex. TSH-releasing analogues were also reviewed in order to further highlight the correlation between maternal and fetal thyroid function and their interaction.

Although maternal TRH crosses the placenta, scientific evidence shows no effect of fetal thyroid function when the hormone is absent. Synthetic TRH crosses the placental barrier, resulting in increased TSH plasma concentrations. Studies in rats, guinea pigs, and human specimens from therapeutic abortions have shown how TRH crosses the placental site into the fetal circulation. This finding, as well as those from other studies, have provided sufficient evidence that TRH, synthetic or not, can stimulate TSH secretion in humans.

Hypothalamic TRH is not superior in terms of placental permeability into extraneural sites of TRH to those of pancreatic origin. The latter observation underlines our limited knowledge of the physiological patterns of this hormone.

With regard to the true role of maternal TRH in fetal thyroid function, the data are scarce. Meanwhile, concerning ATD treatment, the literature shows a certain degree of risk in the development of the fetus associated with their use. No ATD is considered superior to another for treatment of maternal hyperthyroidism. Randomized controlled studies and/or further use of animal models can increase our knowledge in this field.

## Figures and Tables

**Table 1:** Penetrability of various types of TRH and ATDs.

Hormone/Drug	Molecular Weight (g/mol)	Penetrability
Maternal hypothalamic TRH	362.4	+
Exogenous TRH	362.4	+++
Placental TRH	*	+++
Maternal pancreatic TRH	*	+++
Maternal extrahypothalamic TRH	*	++
Iodine	126.9	+++
DIMIT	267.1	+++
PTU	170.2	++
CBZ	186.2	++
MMI	114.1	+++

(+ sufficient, ++ normal, +++ excessive, * no data acquired). (TRH: thyrotropin-releasing hormone; ATDs: antithyroid drugs; DIMIT: 3′,5′-dimethyl,5-isopropyl thyronine; PTU: prophylthiouracil; CBZ: carbimazole; MMI: methimazole).
